# Editorial: Exploring herpesviruses: from biology to disease management and prevention

**DOI:** 10.3389/fimmu.2026.1944802

**Published:** 2026-08-05

**Authors:** Cong Sun, Hua Zhang, Hongbo Wang, Xiao Zhang

**Affiliations:** 1 State Key Laboratory of Oncology in South China, Sun Yat-Sen University Cancer Center, Guangzhou, China; 2 Zhongshan School of Medicine, Sun Yat-sen University, Guangzhou, China; 3 Division of Infectious Disease, Department of Medicine, Brigham and Women’s Hospital, Harvard Medical School, Boston, MA, United States; 4 College of Pharmacy, Chongqing Medical University, Chongqing, China

**Keywords:** clinical biomarker, human herpesviruses, immunopathogenesis, transmission, viral immunity

Human herpesviruses represent an extensive group of persistent pathogens capable of establishing lifelong latent infection following primary host exposure. They trigger a spectrum of immunological disturbances ranging from asymptomatic viral shedding to severe age-related degenerative disorders, central nervous system (CNS) complications and concurrent malignant lesions. This Research Topic features eight peer-reviewed manuscripts, including one methodological framework paper, five original clinical and epidemiological research articles, and two mechanistic review papers. These works collectively unpack the multi-layered crosstalk among herpesviruses, host immunity, population transmission dynamics and clinical disease outcomes, while delivering integrated translational insights for pediatric care, geriatric health management, HIV-associated oncology and infectious disease diagnostics (1, 2).

Del’Olio et al.’s methodological paper lays the methodological groundwork for this Research Topic. The study proposes a two-stage community-engaged research framework named TransmIT, which enables quantitative analysis of cytomegalovirus (CMV) transmission within early education and care (EEC) facilities (Del’Olio et al.). As a prevalent β-herpesvirus, CMV stands as the leading infectious trigger of congenital birth defects and childhood hearing loss, yet the transmission patterns among young children remain insufficiently characterized. This scalable research platform combines digital participant recruitment workflows, standardized saliva-based digital PCR viral load testing and community advisory oversight mechanisms. It facilitates equitable and diverse cohort enrolment, offers a reproducible paradigm for population-level surveillance of herpesviruses in childcare settings, and establishes essential technical support for subsequent vaccine development and intervention trials targeting congenital CMV prevention.

Five original research papers provide empirical evidence across distinct herpesvirus subfamilies and diverse clinical scenarios. Liu et al. draw on the large-scale UK Biobank cohort to identify a previously unreported sex-specific correlation between chromosomally integrated human herpesvirus 6 (ciHHV-6) and sarcopenia. Their findings confirm that DR-only viral integration markedly raises the risk of muscular atrophy in male individuals, with telomere length serving as a critical biological modulator mediating the immune-muscle regulatory axis. Focusing on human herpesvirus 8 (HHV-8, also known as Kaposi sarcoma herpesvirus), Flores-Gonzalez et al. document profound T cell immunosenescence in patients co-infected with HIV and KSHV who develop disseminated Kaposi sarcoma. The team detected expanded populations of senescent CD8+CD57+ T cells, enhanced glycolytic activity mediated by GLUT1, and dysregulated TIM-3/Gal-9 immune checkpoint signaling (Flores-Gonzalez et al.). Their data further demonstrate that combination therapy with valganciclovir can partially reshape senescence-related protease and cytokine expression profiles.

Two original papers focus on varicella-zoster virus (VZV, HHV-3). Recart et al. conduct a 12-year retrospective epidemiological analysis to evaluate the disease burden of herpes zoster (HZ) among privately insured populations in Argentina. The research quantifies age-dependent incidence rates, prevalence of postherpetic neuralgia (PHN) and recurrent zoster incidence, verifying that herpes zoster imposes persistent long-term disease burdens even under universal childhood varicella vaccination schemes. Zhang et al. validate the routine blood platelet-to-lymphocyte ratio (PLR) as a simple, readily accessible inflammatory biomarker for predicting the onset of VZV meningitis and neurological prognosis in patients with acute zoster. This marker serves as a non-invasive auxiliary diagnostic tool that reduces reliance on lumbar puncture procedures. The fifth original study delineates spatiotemporal and age-stratified epidemic trends of varicella in Hangzhou, laying an evidence base for targeted pediatric immunization and outbreak containment strategies (Gu et al.).

Complementing the empirical original studies, two review articles summarize core adaptive immune mechanisms governing herpes simplex virus (HSV) infection. The first review elaborates mucosal chemokine regulation and the formation of tissue-resident memory T (TRM) cells, which act as the primary immune barrier against recurrent HSV reactivation at epithelial surfaces (Lekbach et al.). The second review systematically dissects cell-mediated immune deficiencies that drive HSV-1 encephalitis, outlining pathogenic immune cascades and clinically viable therapeutic directions for CNS herpesvirus infections (Osborne et al.).

Taken together, this Research Topic bridges fundamental immunology, population epidemiology and translational clinical practice. A consistent thematic thread runs through all included works: all human herpesviruses disrupt host immune homeostasis via latent persistence, sustained inflammation and cellular senescence, though viral subfamily, host age, biological sex and co-infection status jointly shape highly heterogeneous clinical manifestations. Key translational takeaways include standardized community surveillance pipelines for CMV, novel geriatric muscle health biomarkers associated with HHV-6, immunosenescence signatures specific to HIV-Kaposi sarcoma patients, and low-cost peripheral blood indicators for VZV-related neurological complications. Across all manuscripts, authors highlight priority directions for future research: sex-stratified investigations of viral immunity, senolytic or antiviral targeted interventions, and expanded equitable cohort studies to enroll underrepresented demographic groups.

We hope this integrated Research Topic fosters interdisciplinary collaboration among immunologists, pediatricians, geriatricians and public health practitioners, so as to alleviate the global disease burden attributable to herpesviruses. We also anticipate the findings compiled herein will act as a foundational reference resource for virologists, clinical immunologists, pediatric infectious disease specialists and population epidemiologists. By integrating basic viral immunology with real-world clinical and population data, this Research Topic links bench-side laboratory discoveries to practical disease prevention strategies.

We express sincere gratitude to all contributing authors, peer reviewers and the editorial team of Frontiers for their efforts in refining this interdisciplinary Research Topic. It is our expectation that these papers will inspire further cross-subfamily research on human herpesviruses, accelerate the development of improved vaccines and immune-targeted therapeutics, and ultimately lower the global morbidity arising from lifelong herpesvirus latent infection.
